# Unconscious and Distinctive Control of Vocal Pitch and Timbre During Altered Auditory Feedback

**DOI:** 10.3389/fpsyg.2020.01224

**Published:** 2020-06-05

**Authors:** Mingdi Xu, Ryosuke O. Tachibana, Kazuo Okanoya, Hiroko Hagiwara, Ryu-ichiro Hashimoto, Fumitaka Homae

**Affiliations:** ^1^Department of Language Sciences, Graduate School of Humanities, Tokyo Metropolitan University, Tokyo, Japan; ^2^Department of Life Sciences, Graduate School of Arts and Sciences, The University of Tokyo, Tokyo, Japan; ^3^Research Center for Language, Brain and Genetics, Tokyo Metropolitan University, Tokyo, Japan

**Keywords:** vocal compensation, formant, fundamental frequency (F0), vocal adjustment, voice production

## Abstract

Vocal control plays a critical role in smooth social communication. Speakers constantly monitor auditory feedback (AF) and make adjustments when their voices deviate from their intentions. Previous studies have shown that when certain acoustic features of the AF are artificially altered, speakers compensate for this alteration in the opposite direction. However, little is known about how the vocal control system implements compensations for alterations of different acoustic features, and associates them with subjective consciousness. The present study investigated whether compensations for the fundamental frequency (F0), which corresponds to perceived pitch, and formants, which contribute to perceived timbre, can be performed unconsciously and independently. Forty native Japanese speakers received two types of altered AF during vowel production that involved shifts of either only the formant frequencies (formant modification; Fm) or both the pitch and formant frequencies (pitch + formant modification; PFm). For each type, three levels of shift (slight, medium, and severe) in both directions (increase or decrease) were used. After the experiment, participants were tested for whether they had perceived a change in the F0 and/or formants. The results showed that (i) only formants were compensated for in the Fm condition, while both the F0 and formants were compensated for in the PFm condition; (ii) the F0 compensation exhibited greater precision than the formant compensation in PFm; and (iii) compensation occurred even when participants misperceived or could not explicitly perceive the alteration in AF. These findings indicate that non-experts can compensate for both formant and F0 modifications in the AF during vocal production, even when the modifications are not explicitly or correctly perceived, which provides further evidence for a dissociation between conscious perception and action in vocal control. We propose that such unconscious control of voice production may enhance rapid adaptation to changing speech environments and facilitate mutual communication.

## Introduction

The voice conveys various paralinguistic information such as one’s physical and emotional states (Nakamura et al., 2001; Belin et al., 2002, 2004; Yovel and Belin, 2013). In social communication, people gauge the emotional states of others and adjust their voices to behave appropriately. More specifically, people tune their voice to better match both listeners’ expectations and their own beliefs about what their voices should sound like (Pardo, 2006; Sitek et al., 2013). Of several acoustic parameters, the following two are accepted as crucial for the social and emotional information of the voice: (1) the fundamental frequency (F0), which corresponds to the perceived pitch of one’s voice and (2) the formants, which refer to the structure of power peaks in the frequency domain in the sound spectrum (Fant, 1960) and contribute to the perceived timbre of one’s voice (Ghazanfar and Rendall, 2008; Baumann and Belin, 2010; Latinus and Belin, 2011; Xu et al., 2013). Previous studies have shown that these two parameters are associated with particular emotions and that regulation of these parameters can determine social and communicative consequences. For instance, tenderness and sadness are primarily associated with a low F0, whereas anger, fear, and happiness are associated with a high F0 (Fonagy, 1978; Ofuka et al., 1994; Banse and Scherer, 1996; Juslin and Laukka, 2003). Moreover, the F0 has been argued to play a role in determining the success or failure of romantic relationships (Weusthoff et al., 2013). As to the formants, the first formant (F1) is lowered when an individual expresses sadness and fear whereas it is raised during expression of happiness and anger (Juslin and Laukka, 2001). Furthermore, smiling leads to increments in formant frequencies, whereas frowning tends to decrease them (Tartter, 1980; Tartter and Braun, 1994).

How do people achieve such intricate adjustments of their own voices? As with other motor control, sensory reafference plays a crucial role in vocal control. An influential model posits that, during speaking, auditory feedback (AF) of the speaker’s own voice is constantly compared with the internally generated signal that predicts auditory reafference (Hickok, 2012). When a mismatch is detected, a comparator sends a signal to the speech control system for subsequent vocal adjustments. Therefore, vocal adjustment is explicitly grounded in the AF in this model. The importance of AF in vocal adjustments is also clear from empirical observations that patients with post-lingual deafness or hearing impairment, who lack normal AF, typically show unstable articulations (Waldstein, 1990; Svirsky and Tobey, 1991; Cowie and Douglas-Cowie, 1992; Perkell et al., 1992; Schenk et al., 2003). These theoretical and empirical findings have motivated numerous experimental studies that examined how the speech control system utilizes online AF to adjust the subsequent articulations.

The real-time perturbation of AF is a commonly adopted experimental protocol used for this purpose in which acoustic parameters of the speaker’s voice are artificially altered and fed back to the speaker while speaking. This procedure aims to cause an acoustic mismatch between the feedback information and the speaker’s planned articulation (Johns et al., 2003; Mitsuya et al., 2011) and to examine changes in the control of articulation in response to the mismatch. It is known that participants typically attempt to adjust their articulations in response to altered feedback without explicit instructions to do so (Siegel and Pick, 1974; Burnett et al., 1998). Such adjustments, in most cases, are in the opposite direction to the manipulation; for instance, when the F0 of the AF is artificially lowered, the speaker tends to raise the pitch of his/her voice. This type of response is called compensation. To date, spontaneous compensation has been demonstrated in several acoustic parameters of the voice including loudness (e.g., Heinks-Maldonaldo and Houde, 2005; Bauer et al., 2006), the F0 (e.g., Burnett et al., 1998; Jones and Munhall, 2000, 2005; Larson et al., 2000; Chen et al., 2007; Behroozmand et al., 2009; Liu et al., 2011), and the formants (e.g., Houde and Jordan, 2002; Purcell and Munhall, 2006b; Villacorta et al., 2007; Munhall et al., 2009; MacDonald et al., 2010, 2011, 2012; Mitsuya et al., 2011). These observations indicate that speakers tend to maintain their own “voice signature” (Belin et al., 2011), which reflects the uniqueness of the speaker’s voice defined by a set of acoustic parameters.

Converging evidence in various motor modalities led us to question whether compensation occurs when speakers do not notice changes in their voice. Previous studies of visuomotor control, using feedback perturbation paradigms, have shown evidence of compensation without conscious perception of the perturbation in various types of movements, including pointing, reaching (Pelisson et al., 1986), and gait control (Quintern et al., 1985). Motor control may utilize explicit and implicit information, and unconscious compensation seems ubiquitous in diverse types of movements. The same observations have been reported in vocal control studies. In previous studies, trained (experienced) singers showed compensatory vocal responses to subtle F0 changes (10–20 cents) in the AF, whereas they were not explicitly conscious of those changes (Griffiths, 2008; Hafke, 2008). Likewise, untrained non-musicians exhibited compensatory vocal responses for such small sizes of F0 changes in the AF, while they were not tested for awareness of the F0 changes (Liu and Larson, 2007). A recent study clearly demonstrated that untrained participants showed compensatory vocal responses to small F0 changes (10 cents) without perceptual detection of any alteration in their modified voice (Scheerer and Jones, 2018). These findings suggest that unconscious compensation for small vocal pitch-shifts can generally be observed, irrespective of expertise in vocal control, although all these studies have used sudden changes of AF in the middle of vocalization as perturbations. This paradigm may elicit sensation of the perturbation itself, in addition to the mismatch between the internally expected AF and the actual one, which may make the AF perturbation more noticeable. Moreover, these previous studies have focused only on pitch, but whether such regulation can be completed without explicit perception for formant control remains unknown.

Given these unresolved issues, the present study aimed to investigate how perception is associated with vocal regulation of two acoustical features: pitch and formants, in a non-expert general population. We introduced AF modification throughout the entire vocalization, instead of introducing it in the middle of vocalization, in order to avoid the possibility that participants notice the abrupt change in AF. Participants were asked to produce vowel sounds continuously while listening to the AF. The AF modification was designed to induce changes at different levels in either the formants only or in both the formants and the pitch. To examine whether participants are able to notice the AF modifications, we played back the original and modified versions of their own voices recorded during the vocalization session, and asked them whether they noticed the difference in pitch and/or formants between these versions. We expected that participants would unconsciously compensate for unperceived and incorrectly perceived modifications in pitch and formants in a distinctive manner.

## Materials and Methods

### Participants

Forty students from Tokyo Metropolitan University (20 females and 20 males) ranging in age from 18 to 26 years (mean = 22.3, *SD* = 1.8) participated in the experiment. All participants were native speakers of Japanese and none reported a history of hearing or speech problems or neurological disorders. They did not have professional vocal experience. Before the experiment, all of the participants signed informed consent forms. The experiment was approved by the Human Subjects Ethics Committee of Tokyo Metropolitan University.

### Procedure

The participants were seated comfortably in a sound-attenuated chamber wearing a set of headphones (ATH-SX1a, Audio-Technica, Corp., Tokyo, Japan) and a microphone (ISOMAX Headset, Countryman Associates, Inc., Menlo Park, CA, United States). They were asked to repeatedly produce, using their normal speaking voice and natural speaking level, the isolated Japanese vowels “a” (used for analysis) or “u” (used as fillers), which were displayed on a gray computer screen at a distance of 80 cm from in front of them (visual angle: 4.3° × 4.3°). Real-time modified AF was fed back to participants via headphones during their vocalization, as used as standard procedure in previous studies (e.g., Liu and Larson, 2007; Hafke, 2008; MacDonald et al., 2011; Mitsuya et al., 2011; Scheerer and Jones, 2018).

**FIGURE 1:**
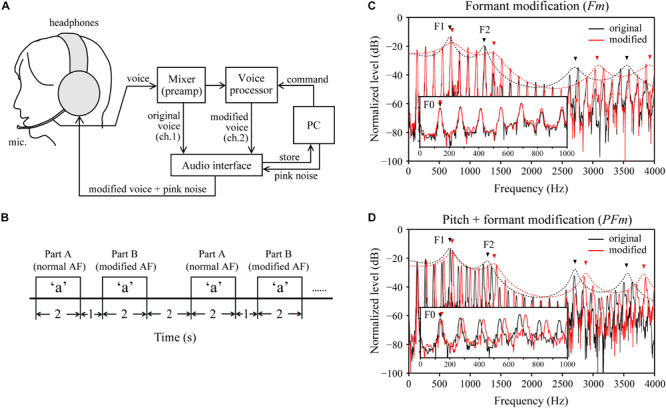
Experimental design. **(A)** A schematic diagram of the voice-changing system used for real time modifications to the auditory feedback (AF). ch: channel. **(B)** Schema for the timing of the task design. AF: auditory feedback. Note that for part B, AF was modified from the onset until the end of vocalization. **(C)** An example of the formant modification (Fm) condition, wherein the formants’ (e.g., the 1st and 2nd formants, F1–F2) frequencies were increased by 200 cents, but the fundamental frequency (F0) was unchanged. **(D)** An example of the pitch + formant modification (PFm) condition, wherein both the F0 and formants frequencies were increased by 100 cents. In both **(C,D)**, black and red lines represent the spectra (solid lines) and spectral envelope (dotted lines) of the original voice and modified voice, respectively, obtained from one male participant during the experiment.

The AF modifications were created using a real-time voice-changing system (Figure 1A). The vocal input collected through the microphone was amplified and divided into two channels using a mixer (Mackie 402-VLZ3, LOUD Technologies, Inc., Woodinville, WA, United States). In one channel, the participant’s original voice was digitally recorded via an audio interface (UA-55, Roland, Corp., Hamamatsu, Japan) at 44100 Hz with a 16-bit sampling rate. In the other channel, the original voice was transmitted to a voice processor (Voice Worksplus, TC Helicon Vocal Technologies, Victoria, BC, Canada) for acoustic modifications. The command signals to alternate modification conditions were sent from a personal computer (PC) to the voice processor through a custom-made set of microcontroller (Arduino Uno R3, Arduino, Somerville, MA, United States) and musical instrument digital interface connectors. Next, the modified voice was recorded onto the PC via the audio interface. Meanwhile, the voice was fed back to the participants over headphones and mixed with masking pink noise, generated by CoolEdit 2000 software (Syntrillium Software, Corp., Scottsdale, AZ, United States). The magnitude of the pink noise was adjusted to maximally minimize the bone conduction of the voice and shield the participants from the air-conducted sound of their unaltered voices.

We asked the participants to keep their voice loudness constant throughout experimental sessions. They used the visual feedback from the loudness indicator on the voice processor to maintain their voice intensity, neither too high nor too low (i.e., all or none of the indicator LED lights on, respectively). As tested with 10 laboratory staff members, the magnitude of the normal speaking voice heard in the earphones was around 75–80 dB sound pressure level (SPL), which was measured at approximately 4-cm distance from the headphones’ diaphragm. We set the intensity of pink noise at around 50 dB SPL after identifying a comfortable level in a preliminary test on 10 laboratory staff members, though the intensity of pink noise has been reported to not affect the vocal responses to pitch-shifts in the AF critically (Burnett et al., 1998). We decreased the level to around 40 dB SPL when several participants reported that the original level was too loud, which is still within the range used in previous modified AF studies (e.g., Burnett et al., 1998; Bauer et al., 2006; Liu and Larson, 2007).

Each trial of the task consists of two parts: parts A and B (Figure 1B). For each part, participants were requested to produce the vowels displayed on a monitor in front of them as soon as possible and lasted for 2 s. We interposed a 1-s interval between parts A and B of a trial. The inter-trial interval was 2 s. The prompted vowels for parts A and B within each trial were always the same. Part A served as the unperturbed baseline for calibration, wherein the participants were fed back with their unaltered voice mixed with pink noise. In part B, participants were fed back with the modified AF, which was introduced from the onset of vowel production and lasted until the end of vocalization. We presumed that the normal AF in part A of one trial could draw a participant’s vowel production back to its normal state after being disturbed in part B of the previous trial. Moreover, how the participants responded to the modified AF (i.e., with compensation or not) could be estimated by comparing the frequency of F0 or formants in part B with that in part A.

Two types of AF modifications, formant modification (Fm) and pitch and formant modification (PFm), each of which had six levels, were used to alter the acoustic parameters of the AF in part B. We used the “Formant” and “Pitch Shift” functions of the voice processor for the Fm and PFm conditions, respectively. In Fm, the vocal spectral envelope was virtually contracted or extended by 3, 6, or 12%, resulting in the increment or decrement of the frequencies of the first formant (F1), and the second formant (F2) by approximately 50, 100, or 200 cents, while the F0 frequency was maintained (Figure 1C). In PFm, the frequencies of the F0 and formants were increased or decreased together by 25, 50, or 100 cents (Figure 1D). In addition to these 12 modified AF conditions, we presented a control condition, wherein the normal AF was presented to the participant in parts A and B of a trial.

For each vowel, there were 10 trials for each of the 13 conditions. The order of the 260 trials was pseudo-randomized with the following two constraints: (1) no more than 3 consecutive trials were of the same vowel production and (2) no consecutive trials were of the same modified AF condition. The presentation of the visual prompts, randomization of trials, and voice recordings were performed with MATLAB R2013b (MathWorks, Natick, MA, United States).

### Subjective Test

After the vocalization experiment, we conducted a subjective test to examine whether participants were conscious of the modifications applied to the AF. For each condition, the participants’ voices recorded in two representative trials during the experiment were used as stimuli. Participants were asked if they could perceive a change in pitch (F0) and/or timbre (formants) between the voices recorded in part A and B of the trial, and answered one of the following four choices: “none,” “pitch,” “timbre,” and “pitch and timbre.” Before starting the subjective test, we verbally explained the meanings of the terms “pitch” and “timbre,” while presenting corresponding sounds of voices that was not the participant’s own voice. Then, we provided a brief practice session for familiarization, in which a set of 13 different trials (1 trial for each condition, different sounds from those used in the actual test) was presented once or twice. We have confirmed that the participants fully understood these terms before starting the actual subjective test.

To assess the patterns of unconscious compensation, we formed two subgroups according to the participants’ subjective responses. One subgroup consisted of participants who responded “none” to the slightest shifts of both directions in Fm (+50 and −50 cents), and the other subgroup contained those who responded “none” to the slightest shifts of both directions in PFm (+25 and −25 cents). The former and latter subgroups included vocal data from 28 and 22 of 40 participants, respectively (Table 1).

**TABLE 1:** Subjective responses in the post-experiment subjective test.

**Response**	**None**	***Fm*: formant modification**						***PFm*: pitch + formant modification**					
	**0**	**200**	**100**	**50**	**−50**	**−100**	**−200**	**100**	**50**	**25**	**−25**	**−50**	**−100**
*“none”*	40	9	22	31	36	22	9	0	6	24	34	17	0
*“pitch”*	0	5	6	3	0	5	6	15	18	8	2	14	19
*“timbre*	0	12	11	6	3	7	11	0	6	7	4	4	2
*“pitch + timbre”*	0	14	1	0	1	6	14	25	10	1	0	5	19

*The numbers represent the numbers of participants who perceived the altered auditory feedback (AF) as only F0-shifted, only formant-shifted, both F0- and formant-shifted, and absent altogether (did not identify any acoustical modification). “none”: participants who did not identify any acoustical modification; “pitch”: participants who perceived the altered AF as only F0-shifted; “timbre”: participants who perceived the altered AF as only formant-shifted; “pitch + timbre”: participants who perceived the altered AF as both F0- and formant-shifted.*

### Data Analysis

#### Extraction of the F0 and Formant Frequencies

The F0 and formant frequencies for the vowel “a” were analyzed for each participant’s original voice to examine any changes in voice production caused by the AF modification, for each trial of each condition. The F0, F1, and F2 in parts A and B were extracted using a speech analysis software Praat 5.3 (Boersma and Weenink, 2013). F0 was calculated for every 10-ms time-step, with a 40-ms time window, by an adapted autocorrelation algorithm. The formant frequencies were estimated for every 10 ms with a time window of 40 ms using an adapted linear predictive coding method (Burg algorithm; see Childers, 1978). First, we estimated the frequencies of a maximum of eight formant candidates; then, F1 and F2 were extracted based on their respective reference frequencies, using a formant-tracking algorithm. The reference frequencies were determined for each participant’s voice in part B of the control condition (i.e., without modified AF). All frequency data were aligned to begin at a voice onset time with sufficient vocal pulses.

#### Pre-processing of Raw Data

The extracted F0 and formant data were pre-processed before further analysis. We eliminated unstable parts at the beginning and end of each utterance caused by instability in the vibration of the vocal folds. To detect these unstable parts, we calculated the time differential of the frequency trajectory for each trial after a four-point smoothing (i.e., moving average), and searched for time points (from onset to 500 ms and from 1000 ms to offset of vocalizations) where the differential value exceeded a predefined threshold. The threshold was defined as differential values 4 *SD* from the middle part (500–1000 ms) of each trial’s frequency trajectory. The detected unstable parts were replaced by null values, which typically constituted 5.9 ± 1.3, 7.6 ± 1.9, and 7.9 ± 2.3% of the original data points for F0, F1, and F2, respectively. Moreover, we removed outlier trials from further analyses when their data appeared to be unreliable due to estimation failure. Outlier trials were identified using two criteria. First, we removed all frequency trajectories whose value exceeded the predefined upper and lower limits at any time point. The upper and lower limits were defined as the third quartile + 3 interquartile range (IQR) and the first quartile – 3 IQR, respectively. Second, any trajectory with a step-like unnatural change exceeding 200 cents between any two consecutive data points within a time range of 500–1500 ms (an interval typically showing a relatively stable utterance, according to visual inspection) was also eliminated. These processes resulted in removals of 0.3 ± 0.8, 3.5 ± 4.7, and 2.3 ± 4.2% of all trials for F0, F1, and F2, respectively.

#### Evaluation of Frequency Changes in F0 and Formants

We estimated the frequency changes in F0 and formants in response to AF modification by the same procedure, using the following three steps. First, frequency data were converted into a logarithmic scale (cent) after dividing each data point of part B (*b*) by the mean frequency within the 500–1500-ms period of part A (ā): 1200 × log2 (*b*/*ā*). Second, we set the beginning part of each vocalization as zero by subtracting the mean value of the first 200 ms (20 points) in each trial to measure only the responses to AF modifications. We defined this subtraction baseline (0–200 ms) by visual inspection of outcomes of the below-described grand averaging, and confirmed that this process appeared not to cause problems for estimating vocal responses to the AF modifications (see Supplementary Figures S1A–D). Third, we removed participant-specific frequency changes that were unrelated to the response to AF modification. For this, a common drift pattern (i.e., trend) in all trajectories for each participant was removed by subtracting the mean of the baseline-subtracted data of all 130 trials. We call this de-trended data the vocal change (VC), which is used to evaluate the vocal response to the AF modifications.

To assess the general tendency, we calculated the grand average of VC obtained from all 40 participants for each condition. Due to a delayed reaction and/or shorter phonation, the duration of vocalization was typically around 1500–2000 ms (see also Supplementary Figure S1E), and hence, we displayed the VC data of only the first 1500 ms. The first 100 ms of the data were somewhat unstable due to the onset of vocalization; thus, we showed the VC trajectory from 100 ms after the onset. We quantified the amount of vocal change for each trial by defining a VC magnitude as the mean value of the latter half (750–1400 ms) of VC data, in which the trajectories fluctuated less and were relatively stable (see Supplementary Figures S1F,G). To examine the relationship between AF modifications and the vocal responses further, we defined a compensation index for each participant. This index was calculated as a sign-inverted slope of a line (linear regression) fitted to the VC magnitudes as a function of frequency changes in the seven AF conditions for each type (i.e., Fm and PFm), including the control condition. It showed a positive value when the participant’s vocal response went in the compensatory direction against the AF modification, and became 1.0 if there was full compensation.

### Statistical Analyses

To confirm whether compensatory responses were prevalent, the number of participants who provided compensatory or following responses for each parameter in the Fm and PFm conditions, determined by the compensation index, were submitted to binomial tests.

To test whether frequency-increased and -decreased AF modifications led to significant differences in VC magnitude data, we conducted discriminant analysis by using IBM SPSS statistics software. This analysis indicates how much two or more data groups differ by identifying a multidimensional classifier that best discriminates between groups at a rate better than chance (Afifi et al., 2003; Cramer, 2003). Here, we aimed to quantify the degree of difference in compensation between two groups of AF modification directionality (increase vs. decrease) for each of the six types of vocal responses (Fm-PFm × F0-F1-F2). For this, we formed a dataset that contained 80 data points (40 participants × increased-decreased groups) in a three-variable space corresponding to the sizes of AF modifications (severe-medium-slight), for each of the six types of vocal responses. We generated the classifier as a linear combination of the three variables using the whole dataset, which best discriminated the two directionalities. The weightings (coefficients) for the combination of three variables were searched to minimize Wilks’s λ (Chan, 2005; Wardak et al., 2011). We reported this minimal λ to assess the accuracy of the estimated model. To examine whether this classification was statistically better than chance (50% for two groups), Press’s Q statistic was calculated to compare the results with the critical value from the chi-square distribution with 1 degree of freedom (Chan, 2005):

$${\mathrm{Press}}^{\prime }\,s\,Q=\frac{{\left(N-\mathrm{nk}\right)}^{2}}{N\,\left(k-1\right)}$$

where k indicates the number of groups for increasing and decreasing shifts (two), N denotes the total sample size (80), and n is the number of data points correctly classified. If Press’s Q value exceeds this critical value (in this case, Q = 6.96), the classification accuracy is significantly better than by chance, thereby indicating that the compensation is statistically significant (significance level: *p* < 0.05 after Bonferroni’s correction). We also performed a one-leave-out cross-validation on the same dataset (Afifi et al., 2003), as follows. We picked one data point and classified it by a classifier model constructed from the rest of the dataset. We repeated this procedure for each of the 80 data points and counted how many data points were classified correctly. The percentage of correctly classified data points was defined as the hit rate, to show classification accuracy.

Next, to examine the effect of modification size, we conducted a series of Friedman tests on the VC magnitude data. Specifically, for each parameter (F0, F1, and F2) and each condition (PFm and Fm), we used modification sizes (±25, ±50, ±100 cents, and 0; i.e., 7 conditions in total for PFm; ±50, ±100, ±200 cents, and 0, 7 conditions in total for Fm) as the within-subject factor of the Friedman test. When significant results were obtained (corrected using Bonferroni’s method), we examined this further using the sign-test (corrected for multiple comparisons using Ryan’s method).

## Results

### Subjective Response

The participants’ subjective responses to the AF modification showed that the PFm conditions were generally more noticeable than the Fm conditions; in other words, fewer participants in the PFm conditions made “none” responses, which indicates that they did not recognize any acoustical modification. In addition, for both the PFm and Fm conditions, it generally became difficult to perceive the AF modifications as their magnitudes decreased; thus, fewer participants recognized the corresponding acoustical modification (Table 1).

Importantly, a number of participants did not recognize any acoustical modification (as illustrated by the “none” responses in Table 1) for the slightest shifts in Fm (± 50 cents) and PFm (± 25 cents). For Fm, 31 and 36 participants responded “none” in the +50 and −50 cents conditions, respectively. Meanwhile, for PFm, 24 and 34 participants responded “none” in the +25 cents and −25 cents conditions, respectively. Next, we gathered vocal data to form two subgroups; one contained data from participants who responded “none” to both directions of the slightest shift in Fm (28 participants), and the other included those who responded “none” to both directions of the slightest shift in PFm (22 participants). To examine the dissociation between perception and production for pitch and formants, the two subgroups were analyzed separately in the following analyses.

### General Tendency in Vocal Response

**FIGURE 2:**
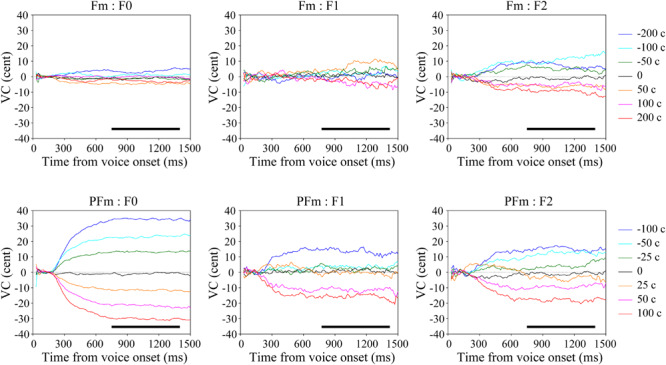
The grand average voice change (VC) of continuous fundamental frequency (F0) and formants (F1–F2) in both types of AF modification conditions. The upper panel shows the results in the formant modification (Fm) conditions and the lower panel shows those in the pitch + formant modification (PFm) conditions. Warm-colored lines represent the frequency-increased conditions and the cold-colored lines represent the frequency-decreased conditions. The black bars show the selected time window (750–1400 ms) with relatively stable vocal responses.

To assess the general tendency shared in all participants, we calculated the grand average of vocal responses over trials and participants for each parameter (F0, F1, and F2) in each condition (Figure 2). For each parameter, the VC was almost zero in the control condition. If the VC in the six-level AF modified conditions diverged into two clusters, on either side of the control, in opposite directions to the frequency shifts applied to the AF, we regarded this pattern to indicate compensation. While there was only F2 compensation in Fm, strong and clear F0 and F2 compensations as well as moderate F1 compensation were observed in PFm. These compensations showed a similar trajectory: the magnitude of compensation increased steadily, reached a plateau at no more than 40% of the size of the frequency shift, and remained stable until the utterance ended. The results from the discriminant analyses confirmed these observations: there were significant F2 compensation in Fm, and significant F0, F1, and F2 compensations in PFm (Table 2).

**TABLE 2:** Summary of the discriminant analysis on all 40 participants.

**AF modification**	**Parameter**	**Wilk’s λ**	**Hit rate (%)**	**Press’s Q**
*Fm*	F0	0.850	62.5	5.0
	F1	0.967	50.0	0.0
	F2	0.674	78.8	26.5*
*PFm*	F0	0.235	96.3	68.5*
	F1	0.745	70.0	12.8*
	F2	0.536	83.8	36.5*

*Hit rate refers to the cross-validated classification accuracy. If the Press’s Q value exceeds the critical value of 6.96 (shown by asterisks), the classification accuracy is better than chance (50%) at a statistically significant level of 0.05 (Bonferroni’s correction performed).*

The results of Friedman tests also showed that there was a significant effect of modification size for F0 [χ^2^(6) = 162.75, *p* < 0.001], F1 [χ^2^(6) = 19.80, *p* = 0.018], and F2 [χ^2^(6) = 53.89, *p* < 0.001] in PFm, and for F0 [χ^2^(6) = 20.35, *p* = 0.014] and F2 [χ^2^(6) = 40.66, *p* < 0.001] in Fm. These results are largely consistent with those of the discriminant analyses. To be conservative, we adopted the results that were significant in both lines of statistical analyses. Namely, there were significant F0, F1, and F2 compensations in PFm, and only significant F2 compensation in Fm.

### Relationship Between Vocal Compensations and AF Modifications

**FIGURE 3:**
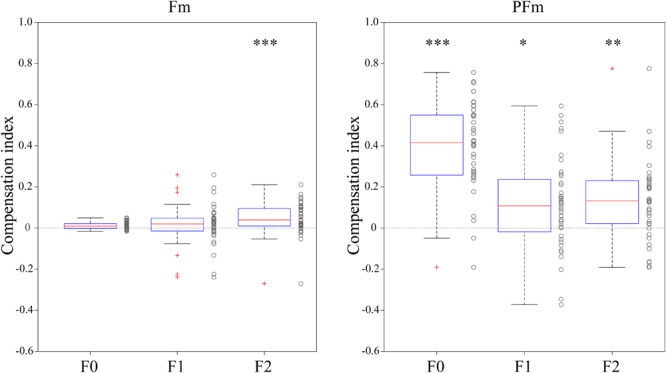
The compensation index for each participant in both types of AF modification conditions. The left panel shows the results in the Fm conditions and the right panel shows those in the PFm conditions. Box plots of compensation indexes were created for each parameter (F0, F1, and F2). The asterisks indicate the statistical significance of the binomial tests (*p < 0.05, **p < 0.01, and ***p < 0.001). The *p-*values of the 6 binomial tests were adjusted using Bonferroni’s correction.

We performed simple linear regressions to quantify the rate of compensation responses against AF modifications as the compensation index of each individual participant. The compensation indices (Figure 3) showed that both Fm and PFm showed trends of compensation and the magnitude of compensation was larger in PFm.

The number of the 40 participants who made compensatory or following responses was computed and submitted to a binomial test to examine whether significantly more participants compensated for the AF modification. It was found that compensatory participants significantly outnumbered following participants for F2 in Fm, and for F0, F1, and F2 in PFm. Over 80% participants made compensatory responses to the F2 (82.5%, *n* = 33) modification in Fm, and the F0 (95%, *n* = 38) and F2 (80%, *n* = 32) modifications in PFm. This result is highly consistent with the discriminant analysis results.

### The F0 Compensation

The F0 compensation was observed in PFm but not in Fm (Figures 2, 3). Particularly, the F0 compensation in PFm exhibited very high precision, in that the magnitudes of the VC in F0 were ranked in order according to the sizes of F0 modifications applied to the AF (Figure 2). Friedman’s test and *post hoc* comparisons validated this observation. Specifically, there were significant differences between all sizes of AF modifications (see Table 3).

**TABLE 3:** Summary of the results of *post hoc* analyses on significant effect of modification size for F0 in PFm (**p < 0.01, ***p < 0.001).

**PFm, F0**	**+100 c**	**+50 c**	**+25 c**	**0**	**−25 c**	**−50 c**	**−100 c**
+100 c		***	**	**	***	***	***
+50 c			**	***	***	***	***
+25 c				**	***	***	***
0					***	***	***
−25 c						**	***
−50 c							**
−100 c							

**FIGURE 4:**
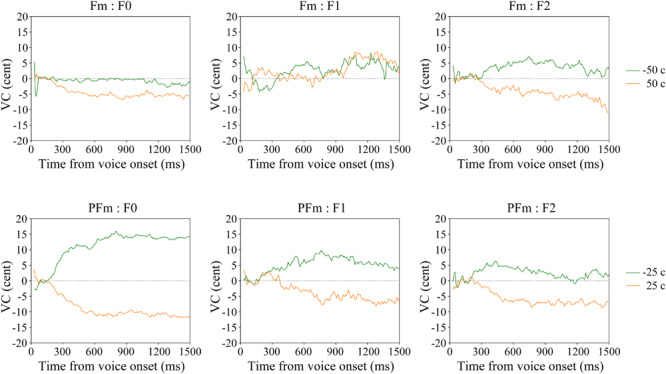
The average VC of continuous F0, F1, and F2 in the Fm and PFm conditions for two subgroups of participants according to their subjective responses.

Interestingly, significant F0 compensation was found even when the frequency shift was so slight (±25 cents) that the participants could hardly recognize it (see the “none” responses in Table 1). To investigate a possible perception-production dissociation for F0, we checked the VCs of 22 participants who failed to recognize any acoustical modification in both +25 and −25 cents in PFm. It was found that they did make significant F0 and formant compensations for these unnoticed F0 and formant modifications (Figure 4 and Table 4).

**TABLE 4:** Summary of the discriminant analysis on the participants who did not identify any acoustical modification in conditions with slightest frequency shift.

**Group**	**Parameter**	**Wilk’s λ**	**Hit rate (%)**	**Press’s Q**
“none” in ± 50 cents of *Fm*	F0	0.925	62.5	3.50
	F1	1.000	41.1	1.77
	F2	0.935	67.9	7.18*
“none” in ± 25 cents of *PFm*	F0	0.341	93.2	32.85*
	F1	0.872	68.2	5.82*
	F2	0.932	63.6	3.27

*“none”: participants who did not recognize any acoustical modification. Twenty-eight participants responded “none” in the ± 50 cents of Fm, while 22 participants responded “none” in the ± 25 cents of PFm. Hit rate refers to the cross-validated classification accuracy. If the Press’s Q value exceeds the critical value of 5.73 (shown by asterisks), the classification accuracy is better than chance (50%) at a statistically significant level of 0.05 (Bonferroni’s correction performed).*

### The F2 Compensation

The F2 compensations in both Fm and PFm showed a pattern similar to the F0 compensation in PFm (Figures 2, 3). Discriminant analysis and Friedman’s test proved significant F2 compensation in both Fm and PFm. The degrees of compensations were almost proportionate to the sizes of modifications. Friedman’s test and *post hoc* comparisons validated this observation (see Table 5, upper panel for PFm and lower panel for Fm). Particularly, for F2 in PFm, significant differences were mainly found between +100 cents and some conditions. To simplify the explanation, the difference between the sizes of modifications in two conditions (e.g., +100 vs. +50 cents) was defined as “distance”; and the larger the difference, the further the distance. Although there was no significant difference between +100 and +50 cents (the condition with nearest distance from +100 cents), significant differences were found between +100 cents and 0, −25 cents, −50 cents, and −100 cents (conditions more distant from +100 cents). Moreover, the level of significance increased as the distance between conditions increased (i.e., *p* < 0.01 for +100 cents vs. 0 and −25 cents; and *p* < 0.001 for +100 cents vs. −50 cents and −100 cents. See the upper panel of Table 5). This indicates a tendency for a gradual difference in the F2 response as a function of modification sizes, although this was not as distinguished as the F0 response in PFm. In addition, a similar tendency was also observed for −100 cents in Fm: the significance level increased as a result of the increase in the distance between conditions (*p* < 0.01 for −100 cents vs. 0; and *p* < 0.001 for −100 cents vs. +50 cents. See the lower panel of Table 5). Interestingly, even though Figure 2 showed larger F2 compensation in −200 and +200 cents (conditions with the largest modification in Fm), the results of *post hoc* analyses did not show the above-mentioned tendency observed for −100 cents in Fm. This may be due to the larger inter-participant variability in −200 and +200 cents. In these two conditions, the modification is so large that the AF notably deviates from one’s own voice. In this situation, inter-participant differences may arise in terms of how much they compensate, and some may have even followed the modification. This possibility warrants further examination in future studies.

**TABLE 5:** Summary of the results of *post hoc* analyses on significant effect of modification size for F2 in both PFm (upper panel) and Fm (lower panel) (*p < 0.05, **p < 0.01, ***p < 0.001).

**PFm, F2**	**+100 c**	**+50 c**	**+25 c**	**0**	**−25 c**	**−50 c**	**−100 c**
+100 c				**	**	***	***
+50 c							*
+25 c							*
0							
−25 c							
−50 c							
−100 c							
**Fm, F2**	**+200 c**	**+100 c**	**+50 c**	**0**	**−50 c**	**−100 c**	**−200 c**
+200 c						*	
+100 c						*	*
+50 c						***	
0						**	
−50 c							
−100 c							
−200 c							

Although few participants recognized the formant modification in Fm (Table 1), their grand average revealed statistically significant F2 compensation (Table 2). To determine whether there was a perception-production dissociation for F2, we examined the VCs of 28 participants who made “none” responses in both +50 cents and −50 cents in Fm, wherein the formant frequency modification was slightest. It showed that these participants made significant F2 compensation for formant modifications that they could not notice (Figure 4 and Table 4).

### The F1 Compensation

The F1 responses showed some compensation in PFm but not in Fm (Figures 2, 3). Discriminant analysis and Friedman’s test validated significant F1 compensation in PFm but not in Fm, while *post hoc* analyses revealed no significant difference between any modification sizes.

## Discussion

The present study investigated whether the vocal control system implements F0 and formant compensation when participants engaging in vowel production are not explicitly conscious of acoustic changes in the AF. Furthermore, if so, the present study investigates exactly how this compensation may be implemented. Our findings demonstrated that participants involuntarily compensate for F0 and formant modifications in such cases. The grand average results of voice production revealed that significant F0 and F2 compensation was found in the PFm and Fm conditions, respectively, although the majority of participants did not perceive these frequency shifts. When we focused on the conditions with the slightest modifications (± 50 cents of Fm and ± 25 cents of PFm), we found that participants who did not perceive any changes in their own voice in the Fm condition compensated for the F2 modification, and similarly those who did not recognize any alteration in their own voice in the PFm conditions made significant F0 compensation. These findings demonstrate implicit control of the production of pitch and formants without explicit perception.

### Compensation for Ambiguous AF Perturbations

The subjective test indicates that participants made compensation for AF modifications that they could not perceive or misperceived. In addition, it can be confirmed from Table 1 that (1) there was no participant that answered “none” to the ± 100 cents of PFm and that, (2) for both Fm and PFm, the number of participants who responded “none” increased as the size of modifications in AF decreased. These findings demonstrate that, rather than being biased to choose “none,” the participants fully understood the rules of the subjective test and their perceptions were correctly reflected in their subjective responses. The compensation for pitch modifications as small as 25 cents is consistent with several previous studies that investigated voice responses of the F0 using pitch-shifted AF (Burnett et al., 1997, 1998; Larson et al., 2001; Liu and Larson, 2007). However, these studies did not directly examine whether the 25-cent shifts were below or above the perceptual threshold of participants. In the present study, the divergence between the participants’ perception and their vocal compensatory responses implies a dissociation between the ability to recognize changes in one’s own voice and the ability to control articulation. This dissociation has been found between perception and production of pitch (F0) in previous studies (Bradshaw and McHenry, 2005; Dalla Bella et al., 2007, 2009, 2011; Pfordresher and Brown, 2007; Hafke, 2008; Loui et al., 2008; Moore et al., 2008; Hutchins and Peretz, 2012). These studies investigated two groups of participants at both ends of the musical spectrum: those with congenital amusia and trained musicians. Several studies of the singing voice have found that some individuals with amusia have spared ability to reproduce musical intervals that they cannot consciously discriminate, although it is worse than that of a control group (Loui et al., 2008; Dalla Bella et al., 2009; Hutchins and Peretz, 2012). Hafke (2008) suggested that trained singers can adjust their voice to compensate for pitch shifts in their own voice that they are unable to distinguish. Our results demonstrated that the dissociation between pitch perception and pitch production also occurs in most participants who are not trained musicians.

A recent study by Scheerer and Jones (2018) also reported that their untrained participants compensated for pitch-shifts as small as 10 cents, while their sensitivity, indexed by d-prime, revealed that they detected pitch-shift of 15 cents and larger. Although there was a slight difference between the size of pitch-shift that elicited vocal compensation and perceptual awareness, the authors suggested that the inherent variability in vocal production might have caused the participants to be less certain of whether they heard a pitch-shift during vocalization. The present study did not use modifications smaller than 25 cents, and thus we cannot fully compare our results with this study on vocal responses, but our vocal result on 25 cents in PFm was highly consistent with this previous study. With respect to the subjective results, we used a post-experiment test on consciousness of frequency modifications, which is comparable to the listening condition used by Scheerer and Jones (2018). Their results showed that participants can only perceive 25- and 40-cents pitch-shifts in the listening condition, but could perceive pitch-shifts as small as 15−40 cents in the vocalization condition. In comparison, we found that less than half of participants (Table 1) were able to detect a 25-cent pitch-shift. This discrepancy may be partially because we used a more difficult subjective test for the participants to detect modifications. Specifically, vocal samples recorded during the vocalization task were presented to the participants with the same settings as in the vocalization task. Participants were asked to tell whether they could hear any difference between part A and part B of a trial, which were separated by an interval of 1 s. In part A, the participant’s voice was unmodified and served as the reference for comparison; in part B, the participant’s voice was modified throughout the vocalization period. In contrast, Scheerer and Jones (2018) used a paradigm in which sudden perturbations were introduced in the middle of vocalization, and any period immediately prior to the perturbation served as a reference, making the sudden perturbations more noticeable than ours.

The vocal responses of the two subgroups of participants, who gave “none” responses in Table 1, revealed that the unconscious compensation for AF modification is highly specific to its content. In particular, when there was only formant modification in Fm, compensation was found to be significant for the formant (F2) but not for F0, while when there were both pitch and formant modifications in PFm, the participants compensated significantly for both F0 and the formant (F1; Figure 4 and Table 4). These results, together with the largely consistent findings in the grand average data (Figures 2, 3 and Table 2), indicate that the vocal control system is able to execute flexible compensation for modification in voice; especially, formant compensation can be independent or in parallel with pitch compensation. As reasoned by Burnett et al. (1997), who found that participants responded to pitch shifts by changing the F0 independently of loudness, the neural system for vocal control may have separate channels for various acoustic features.

Our findings extended the perception-production dissociation to compensations in AF conditions that participants noticed the changes. Very few participants correctly recognized the formant modification in Fm as a change in the timbre (formants) of their voices, even when the frequency change was as large as ± 200 cents (Table 1). In particular, most participants reported perceiving some abnormality in their voices but were not sure whether it was a change in pitch or timbre, as shown in the number of participants who responded “pitch” or “pitch + timbre” instead of “timbre.” Despite this uncertainty, the participants did not adjust their pitch in response to falsely detected F0 modifications, but did fine-tune their F2 to compensate for the real formant modifications (Figures 2, 3). Such divergence between incomplete recognition and appropriate vocal response suggests that the vocal control system functions with high accuracy and is able to implement compensation even when the modification is incorrectly registered by participants.

We found significant F2, but not F1 compensation in Fm, for both the grand average and the “None” response subgroup (Figures 2, 4, upper panel), while F1 compensation had been found in previous formant perturbation studies (Houde and Jordan, 1998, 2002; Purcell and Munhall, 2006a, b; Villacorta et al., 2007; MacDonald et al., 2010, 2011, 2012; Mitsuya et al., 2011). This discrepancy may be due to the size of frequency modifications applied to F1. Previous studies used much larger formant-shifts than those used in our study. Houde and Jordan (1998, 2002) used F1 and F2 shifts of two vowel categories. Other studies used an F1 modification of either one vowel category (e.g., shifting the English vowel /ε/ to /æ/ or /I/), yielding a frequency change of around 135 Hz (Purcell and Munhall, 2006b), or up to 200 Hz (Purcell and Munhall, 2006a; Villacorta et al., 2007; MacDonald et al., 2010, 2011, 2012; Mitsuya et al., 2011). In contrast, our formant shifts of 25, 50, 100, and 200 cents changed the F1 frequency (738.6 Hz on average) of the Japanese vowel “a” for only about 11, 22, 44, and 90 Hz. One study reported that the least frequency shift that can induce significant F1 compensation was 76 Hz (Purcell and Munhall, 2006a).

Interestingly, when formants were modified simultaneously with pitch, as in PFm, the F1 compensation was significant for both the grand average and the “None” response subgroup (Figures 2, 4, lower panel). This phenomenon led us to suppose that F1 compensation possibly follows F0 compensation. In addition, from the grand averaged results (Figure 2), F2 compensation also seems to be generally amplified in the PFm conditions, as compared to the Fm conditions, although the size of formant modification in PFm was only half of that in Fm. We propose two possible interpretations of this phenomenon. On the one hand, when the vocal control system attempts to correct for pitch errors, correction for formant errors may be facilitated. On the other hand, the vocal control system possibly executes formant compensations to facilitate pitch compensation. These influences between pitch and formant compensation might appear when untrained, naïve participants control their voices. Moreover, we also conjecture that, when F1 and F2 were modified at the same ratio, F2 compensation would take a priority. Among the Japanese vowels, vowel “a” has the highest F1 frequency and a middle-level F2 frequency. This may have led to the discrepancy between the compensatory vocal responses in F1 and F2 in the Fm conditions of the present study. Using another Japanese vowel, “e,” which has middle-level frequencies for both F1 and F2, may help to assess this possibility. Validation of these speculations is not the focus of the present study, but this should be investigated in future studies.

Vocal responses within the present chosen time window have been suggested to reflect voluntary adjustments of vocal responses to pitch perturbations (Burnett et al., 1998; Hain et al., 2000). Its underlying neural mechanisms are suggested to be distinct (Burnett et al., 1998; Hain et al., 2000; Zarate et al., 2010; Patel et al., 2014) from vocal responses within a relatively early window of 50–400 ms, which have been investigated in several studies focusing on the involuntary pitch-shift reflex (Chen et al., 2007; Liu et al., 2010; Scheerer et al., 2013). However, it has also been reported that, in comparison to the late response, the early response is more susceptible to various factors, such as the vocal F0 (Liu et al., 2010) and the vocal control strategy (Hain et al., 2000). Therefore, the present study chose to focus on this presumably more stable late vocal response.

Our data did not show a clear tendency of the “early” component (50–150 ms) of vocal response in F0 (see Supplementary Figures S1F,G), which has been reported in previous studies (e.g., Burnett et al., 1998; Hain et al., 2000). This suggests a possibility that this previously reported early component is specific to the experimental paradigm in which sudden pitch shifts in the middle of vocalization were used, and it is therefore not necessarily observed in our constant-shift paradigm. This issue should be investigated in future studies.

Note that the subjective test was performed after completion of the vocalization sessions, because we were concerned that a subjective test immediately after each trial may influence the participant’s vocal behavior in the following trial. Since the neural mechanisms of monitoring changes in recorded self-voice and online vocalization may differ, a carefully designed subjective test, immediately after each trial, will be performed in our future experiments.

### Trajectory of Compensation

As noted above, the F0 and F2 compensations showed a similar trajectory: the magnitude of the compensation increased gradually before plateauing and remaining stable until the vocalization ended (Figure 2). The plateau pattern observed in our study is in line with the findings of studies that used relatively longer intervals of pitch shifts (>1000 ms), such as Burnett et al. (1997) and Patel et al. (2014); however, neither study discussed this pattern. We interpret this continuing compensation as indicative of the constant and robust functioning of the vocal control system, as long as disparity between ongoing vocalization and AF exists, which stabilizes vocal characteristics at the desired level and produces a plateau.

The Directions Into Velocities of Articulators (DIVA) model (Guenther, 2006; Villacorta et al., 2007; Tourville et al., 2008) may explain this pattern of compensation. The model suggests that a feed-forward control subsystem and a sensory feedback control subsystem, which includes AF and somatosensory feedback (SF), work in parallel to issue appropriate motor commands to produce desired vocal sounds. In normal speech conditions, highly practiced feed-forward control dominates the motor command signals, and neither AF nor SF contributes due to accurate feed-forward commands. Therefore, there is no mismatch between expected and real sensory feedback. However, artificial perturbation of AF and/or SF would break the balance. Such mismatch between expected and actual sensory consequences results in error signals and causes the feedback-based motor commands to increase and significantly influence the output motor commands for vocal error correction. The initial gradual increase in compensation size possibly reflects the exploratory fine-tuning process of the DIVA model that edits the output motor commands to adjust for the vocal errors.

The incomplete compensation in each AF modification condition, and its sustained saturation (i.e., the plateau) at no more than 40% of the modification, may be due to the influence of the unmodified SF in the present modification method. According to the DIVA model, as the feedforward commands, which incorporate the AF-based corrective commands, change to compensate for the auditory errors, somatosensory errors begin to arise because the AF-based compensation moves the production away from the expected somatosensory target. Correction commands based on SF errors, driven by the AF-based correction, also act and repel changes in the feedforward commands, limiting the extent of AF-based compensation. In our data, the compensation produced for the largest AF modification (e.g., ± 100 cents in PFm) was sufficiently large to completely overcome the smaller modification (e.g., ± 25 cents in PFm). However, it does not support the assumption that the vocal control system only makes a partial compensation when the AF alone is modified because the AF and SF jointly govern the system and some form of multi-modality signal integration (i.e., weighting) must occur. Changing the relative weighting of a specific kind of sensory feedback would cause the vocal control system to design a new corrective strategy to manage a trade-off (MacDonald et al., 2010) between AF-based and SF-based correction commands. When a balance is struck, it would maintain the strategy to stabilize vocal characteristics within a desired range, thereby producing a plateau. The AF and SF weightings may vary for the production of different vowels, and individual discrepancies in such weightings may account for the large inter-participant variance in vocal compensation observed in the present and previous studies (Burnett et al., 1998; Purcell and Munhall, 2006a, b; Munhall et al., 2009). To what degree the vocal control system weighs the feedback from each modality is unknown, while evidence from audio-visual interactions in vocal control supports the idea that multisensory information can be integrated flexibly to optimize vocal control (Larson et al., 2008; Therrien et al., 2012). Considering that somatosensory inputs from facial skin affect the processing of speech sounds (Ito et al., 2009), it would be of interest in future to examine the movements of supraglottal articulators (e.g., jaw and tongue) to test for any possible interaction between auditory and proprioceptive feedback.

### High Precision of F0 Compensation in PFm

It is striking that the magnitudes of the F0 compensation in PFm were ranked as a function of the sizes of pitch modifications, as seen in Figure 2. Specifically, in either direction of AF modification, the greater the pitch modification, the larger the F0 compensation. The difference in compensation magnitudes between all pairs of conditions was significant, suggesting that the vocal control system not only detects errors in the F0 but also gauges their relative size and adjusts it accordingly.

This observation was consistent with two previous studies using small-size pitch-shifts (Liu and Larson, 2007; Scheerer and Jones, 2018), although these studies either used bidirectional pitch-shifts (± 10, 20, 30, 40, and 50 cents) but averaged responses to upward and downward perturbations (Liu and Larson, 2007), or used only downward (−5, −10, −15, −20, −25, −30, and −40 cents) pitch-shifts (Scheerer and Jones, 2018). This intriguing relationship between the stimuli and responses differs from the results of some previous studies (Burnett et al., 1997, 1998; Larson et al., 2001; Chen et al., 2007). The discrepancy might be due to various methodological factors. For instance, although the sizes of pitch shift in these studies overlapped with ours, the onset and duration of the shifts differed, as did the number of participants and averaged trials for each condition. The present study introduced pitch shifts to the AF immediately after the participant’s voice onset, while these past studies used pitch shifts with unexpected timing. Although immediate AF modification may increase expectation and habituation effects, our strict randomization of the 13 conditions and use of fillers involving the vowel “u” might have minimized these considerations. Chen et al. (2007) used 200 ms of unexpected pitch shifts (50, 100, and 200 cents) and the vocal control system may not always recognize such short pitch shifts. Larson et al. (2001) employed a longer duration (>2500 ms) of unexpected pitch shifts (25, 100, and 200 cents) but they used different groups of participants for the three pitch-shift magnitudes, which reduces comparability of this study with ours. This was also true for Burnett et al. (1997), who applied pitch shifts at around 1500 ms and used different groups across six sizes of pitch shift (25, 50, 100, 150, 200, and 300 cents). In fact, they reported that some participants showed systematic changes as a function of pitch-shift size, but the wide inter-participant variability may have obscured any statistically significant difference. In a later study, Burnett et al. (1998) systematically investigated the relationship between the magnitude of the compensation and pitch-shift size (25, 50, 100, 150, 200, 250, and 300 cents). However, the small number of averaged trials (<10) and participants (10) for each size may account for why they did not find any effect of stimulus size on response magnitude.

### Limitations

First, the present study used a separate session of subjective testing after the completion of the whole experiment, in which the voice monitoring condition may be different from that during the online voice production task. A subjective test for each trial immediately after vocalizing while hearing the altered AF may provide more convincing results and can be adopted in future studies. However, we are afraid that this type of test requires more time to execute, and that participants’ judgments may affect their performance in the following trial. Second, because the voice production task of the present study only used vowel production, as what has been done in the previous literature, care must be taken to generalize the findings to more complicated cases of speech production, such as word and sentence production.

## Conclusion

Unconscious adjustment for vocal errors is essential for people with or without professional vocal experience to maintain their voices within a normal range, and this mechanism may not be restricted to pitch because it is similar for formants as well. Our data showed that untrained participants compensated for AF modifications that were incorrectly perceived. These findings lead to a prediction of separate perception-related and production-related representations for self-voice processing, and the latter representation can, at least partly, function without being mediated by the former. The unconscious control of voice production may enhance rapid adaptation to changing speech environments and facilitate mutual communication. Speaker-related factors, including native language, strategy, and personal traits, such as self-consciousness, may also account for the present findings and should be systematically explored in future studies.

## Data Availability Statement

The datasets generated for this study are available on request to the corresponding author.

## Ethics Statement

The studies involving human participants were reviewed and approved by Human Subjects Ethics Committee of Tokyo Metropolitan University. The patients/participants provided their written informed consent to participate in this study.

## Author Contributions

MX and RT contributed to the design of the study, programming and conduct of the experiment, data analysis, interpretation of data, and writing the manuscript. KO and HH supervised the study. RH and FH contributed to the design of the study, interpretation of data, and revision of the manuscript.

## Conflict of Interest

The authors declare that the research was conducted in the absence of any commercial or financial relationships that could be construed as a potential conflict of interest.
