# Supplementary material for: Unconscious and Distinctive Control of Vocal Pitch and Timbre During Altered Auditory Feedback
Source: Front Psychol. 2020 Jun 5;11:1224. doi: 10.3389/fpsyg.2020.01224 (PMC7294928; doi:10.3389/fpsyg.2020.01224)
Supplement: Supplementary file 1 [file Table_1.DOCX]

**Supplementary material**

**General tendency of vocal responses**

To assess the general tendency of the voice change (VC), we calculated the grand averages of the F0 data in the PFm conditions in several different ways. First, we aimed to search for the appropriate period to be used as the subtraction baseline, which was used to set the beginning part of VC around zero in each trial. For this aim, we systematically changed the period among the first 150, 200, 250, or 300 ms, and calculated the grand averaged traces of F0 in the PFm conditions of +100 and -100 cents (Figure S1A‒D). The results showed that the major vocal (compensative) responses start 200 ms after the vocalization onsets, indicating that our procedure using the first 200 ms period did not induce a problem of mixing the early vocal responses into the subtraction base. Then, we also assessed the general tendency of vocal duration to determine the time range for analysis. We confirmed from the results that participants vocalized for around 2 s in almost all trials, while it would be safe to use only the first 1.5 s of data (Figure S1E). Finally, we searched for the stable part (or the plateau) of vocal responses to define an appropriate time window for evaluating the amount of compensative vocal responses (the VC magnitude). For this purpose, we calculated the absolute value of mean VC in the +100 and -100 cents conditions of PFm for each participant, and then plotted the grand average and standard errors among participants (Figure S1F). Moreover, the time derivative of VC data was also computed to visualize how much the vocal response fluctuated at each time point (Figure S1G). These results demonstrated that the major component of vocal responses appears around 200‒400 ms, and the time window used in the present study (750‒1400 ms) was appropriate for assessing the amount of vocal responses.


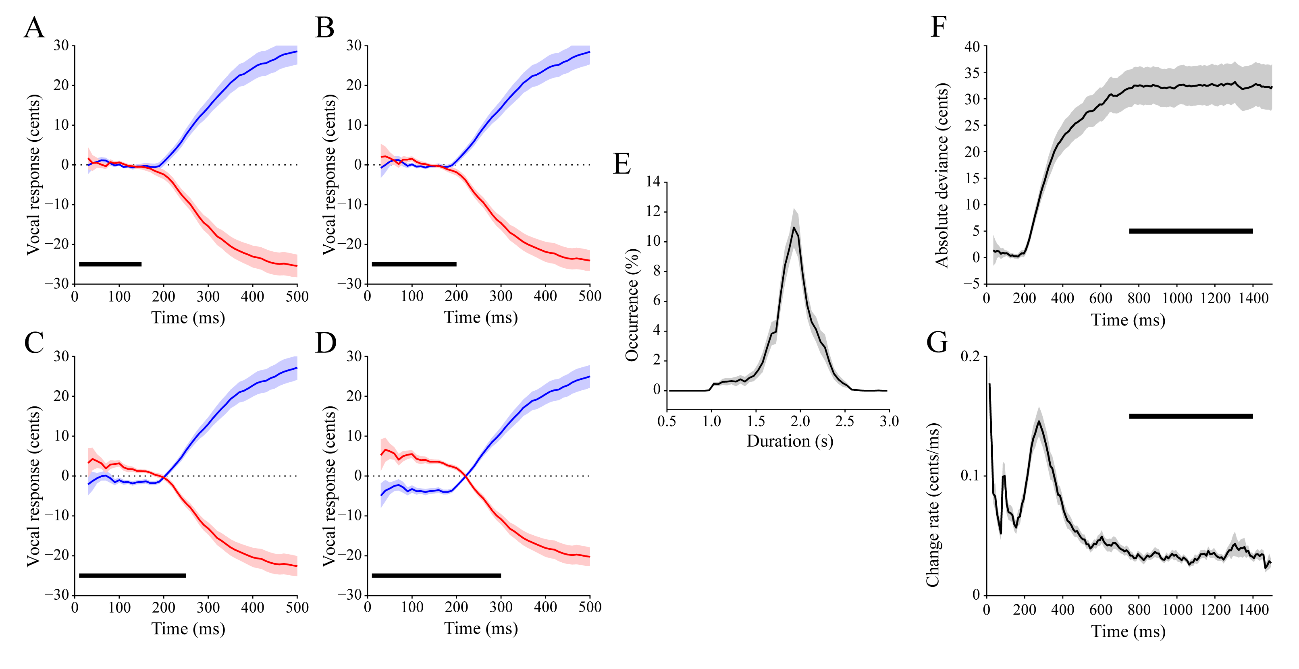


Figure S1. General tendency of vocal change (VC). A‒D. Mean ± SE of vocal responses in the +100 and ‒100 cents (red and blue lines, respectively) of PFm conditions. The average value of the first 150, 200, 250, or 300 ms (shown by black horizontal bars) was used as the subtraction baseline to normalize each vocalization. The hatched area shows the standard error (n = 40). E. Occurrence (in percentage) of voice durations in part B. F. Absolute value of F0 deviance from the baseline, showing that the vocal F0 is almost flat (a plateau) during the evaluation period (between 750 ms and 1400 ms, depicted as a black horizontal bar). G. F0 change rate (i.e., velocity) in cents per milliseconds, showing that the major component of vocal response appears around 200‒400 ms. In all panels of E‒G, the black line indicates the grand average among participants, and the hatched gray area shows the standard error (n = 40).
